# Cleavage‐Responsive Biofactory T Cells Suppress Infectious Diseases‐Associated Hypercytokinemia

**DOI:** 10.1002/advs.202201883

**Published:** 2022-06-25

**Authors:** Hyelim Kim, Boram Son, Eun U Seo, Miji Kwon, June Hong Ahn, Heungsoo Shin, Gyu Yong Song, Eun Ji Park, Dong Hee Na, Seung‐Woo Cho, Hong Nam Kim, Hee Ho Park, Wonhwa Lee

**Affiliations:** ^1^ Brain Science Institute Korea Institute of Science and Technology (KIST) Seoul 02792 Republic of Korea; ^2^ Department of Biotechnology Yonsei University Seoul 03722 Republic of Korea; ^3^ Department of Bioengineering Hanyang University Seoul 04763 Republic of Korea; ^4^ Division of Bio‐Medical Science and Technology (KIST School) Korea University of Science and Technology (UST) Seoul 02792 Republic of Korea; ^5^ Department of Smart Health Science and Technology Kangwon National University Chuncheon 24341 Republic of Korea; ^6^ Division of Pulmonology and Allergy Department of Internal Medicine College of Medicine Yeungnam University and Regional Center for Respiratory Diseases Yeungnam University Medical Center Daegu 42415 Republic of Korea; ^7^ College of Pharmacy Chungnam National University Daejeon 34134 Republic of Korea; ^8^ AREZ Co. Ltd Daejeon 34134 Republic of Korea; ^9^ D&D Pharmatech Seongnam 13486 Republic of Korea; ^10^ College of Pharmacy Chung‐Ang University Seoul 06974 Republic of Korea; ^11^ School of Mechanical Engineering Yonsei University Seoul 03722 Republic of Korea; ^12^ Yonsei‐KIST Convergence Research Institute Yonsei University Seoul 03722 Republic of Korea; ^13^ BK21 FOUR Education and Research Group for Biopharmaceutical Innovation Leader Hanyang University Seoul 04763 Republic of Korea; ^14^ Research Institute for Convergence of Basic Science Hanyang University Seoul 04763 Republic of Korea; ^15^ Department of Chemistry Sungkyunkwan University Suwon 16419 Republic of Korea

**Keywords:** COVID‐19, engineered blood vessel, engineered T cell, hypercytokinemia, infectious disease

## Abstract

Severe infectious diseases, such as coronavirus disease 2019 (COVID‐19), can induce hypercytokinemia and multiple organ failure. In spite of the growing demand for peptide therapeutics against infectious diseases, current small molecule‐based strategies still require frequent administration due to limited half‐life and enzymatic digestion in blood. To overcome this challenge, a strategy to continuously express multi‐level therapeutic peptide drugs on the surface of immune cells, is established. Here, chimeric T cells stably expressing therapeutic peptides are presented for treatment of severe infectious diseases. Using lentiviral system, T cells are engineered to express multi‐level therapeutic peptides with matrix metallopeptidases‐ (MMP‐) and tumor necrosis factor alpha converting enzyme‐ (TACE‐) responsive cleavage sites on the surface. The enzymatic cleavage releases γ‐carboxyglutamic acid of protein C (PC‐Gla) domain and thrombin receptor agonist peptide (TRAP), which activate endothelial protein C receptor (EPCR) and protease‐activated receptor‐1 (PAR‐1), respectively. These chimeric T cells prevent vascular damage in tissue‐engineered blood vessel and suppress hypercytokinemia and lung tissue damages in vivo, demonstrating promise for use of engineered T cells against sepsis and other infectious‐related diseases.

## Graphical abstract

A chimeric T cell that continuously expresses multi‐level therapeutic peptide drugs on the surface of the immune cell is presented. These chimeric T cells prevent vascular damage in the tissue‐engineered blood vessels and suppress hypercytokinemia and lung tissue damages in vivo, demonstrating promise for use of engineered T cells against infectious diseases.

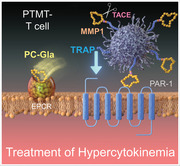

## 1 Introduction

The timely administration of therapeutics is crucial for the treatment of infectious diseases such as coronavirus disease 2019 (COVID‐19). Since the emergence of COVID‐19 at the end of 2019, pharmaceutical companies and researchers have been persistently devoted to developing therapeutics for emerging infectious diseases. The situation is still highly fluid and the infectious disease‐related numbers continue to rise due to breakthrough infections even after vaccination.^[^ ^1^ ^]^ In spite of the efforts and even with the advances in therapies and intensive care, currently, there are no treatment strategies nor effective preventative measures against, especially for, severely progressed infectious diseases. Among infection‐related diseases, sepsis is a systemic inflammatory syndrome caused by acute microbial infection, which leads to over activation of the immune system. Sepsis is a life‐threatening disease that has become a major social issue because it is still one of the leading causes of death in intensive care units.^[^ ^2^ ^]^ Currently, there are no treatments available for sepsis and thus, development of an effective alternative therapeutic strategy is urgently needed. Hence, patients in severe stage of the disease are faced with the dangers of cytokine storm and thus, protection of blood vessel is of vital importance due to leakage of inflammatory cytokines and endotoxins which can cause detrimental effects, such as cytokine release syndrome (CRS) and tissues damages.^[^ ^3^ ^]^ Numerous studies have shown that cytokine storm and tissue damages are significant barriers that need to be controlled in critical patients.^[^ ^4^ ^]^ Even after the successful treatment of the infectious disease, patients may not fully recover from the tissue damage, and the severity of the disease may progress further to multiple organ failure and death.^[^ ^5^ ^]^ As acute complications and death casualties begin with the virus infection, a new form of treatment strategy is urgently required to resolve the rapid infection‐related inflammatory responses.

Peptide‐based therapeutics, also termed biologically active (bioactive) peptides or peptide drugs, have a long successful history in contribution toward human health from the advent of insulin therapy in the 1920s.^[^ ^6^ ^]^ Peptides have been investigated across a wide range of therapeutic spectrums. Currently, there are over 60 peptide‐based therapeutics that have been approved and the number continues to rise as over 150 peptides are in active development today awaiting to enter clinical development. Although the peptide‐based therapeutics have shown promising effects in signaling regulation of immune cells and protection of vascular integrity, their application in vivo is limited due to the short plasma half‐life of peptides in blood circulation.^[^ ^7^ ^]^ The blood circulating therapeutic peptides are prone to enzymatic digestion, which causes loss in their stability and activity. Recombinant activated protein C (APC, trade name Xigris, Eli Lilly) as severe sepsis treatment, with known anti‐coagulant, anti‐inflammatory, and fibrinolytic properties,^[^ ^8^ ^]^ has a very short half‐life of ≈30 min in citrated blood and 15–18 min in whole blood.^[^ ^9^ ^]^ APC is rapidly eliminated from the blood and thus, timely or frequent administration is required to achieve a steady‐state dose. In 2011, The U.S. Food and Drug Administration (FDA) announced withdrawal of Xigris from the market due to failure to show a survival benefit.^[^ ^10^ ^]^ Many researchers explained that the reason for the low efficacy of APC is due to receptor cleavage, such as endothelial protein C receptor (EPCR),^[^ ^11^ ^]^ and protease‐activated receptor‐1 (PAR‐1),^[^ ^12^ ^]^ by released cytokines in severe inflammation. This enables the tumor necrosis factor alpha converting enzyme (TACE) and matrix metallopeptidases 1 (MMP1) proteases to cleave EPCR and PAR‐1.^[^ ^11^, ^13^ ^]^

The cytoprotective effects of APC are reliant on the interaction between the *γ*‐carboxyglutamic acid (Gla) domain of APC and EPCR and the activation of PAR‐1.^[^ ^8^, ^14^ ^]^ This induces cytoprotective signaling responses, such as anti‐inflammation and barrier protection.^[^ ^8^, ^11^ ^]^ Subsequently, this induces cytoprotective signaling responses, such as anti‐inflammation and barrier protection. However, it has been reported that thrombin has at least three orders of magnitude higher catalytic efficiency to cleave PAR‐1 than APC,^[^ ^15^ ^]^ and thrombin receptor agonist peptide (TRAP) activates PAR‐1 by mimicking the effect of thrombin in human endothelial cells.^[^ ^16^ ^]^ Previously, it was demonstrated that upon PC binding to EPCR, it recruits PAR‐1 and initiates PAR‐1‐dependent signaling by thrombin or TRAP switches, thereby shifting from a proinflammatory state to a cytoprotective response in endothelial cells.^[^ ^14^, ^17^ ^]^ This has led us to hypothesize that specific peptides stably expressing from a carrier to simultaneous occupation of both EPCR and PAR‐1 could downregulate and suppress hypercytokinemia with no complications.

Here, we sought to determine whether T cells engineered to express PC–Gla–TACE–MMP–TRAP (PTMT) peptides on the surface and systemic release of therapeutics could be used as a possible treatment therapy for severe infectious diseases. We analyzed cleavage of PC–Gla and TRAP upon the exposure to infectious disease patients’ plasma. We showed that PTMT–T cell protects EPCR shedding in inflammatory conditions and also improves survival in a CLP‐induced septic mouse model. Currently, there are no FDA‐approved therapeutic peptides available for treating patients with severe infectious disease. Engineering chimeric T cells to stably express and systemically release therapeutic peptides is a novel strategy for the treatment of patients with COVID‐19 and other severe infectious diseases, and may ultimately contribute to the development of next generation therapeutic peptide therapy.

## 2 Results

### 2.1 Design of Engineered Therapeutic Peptide‐Expressing Chimeric T Cell

**Figure 1:**
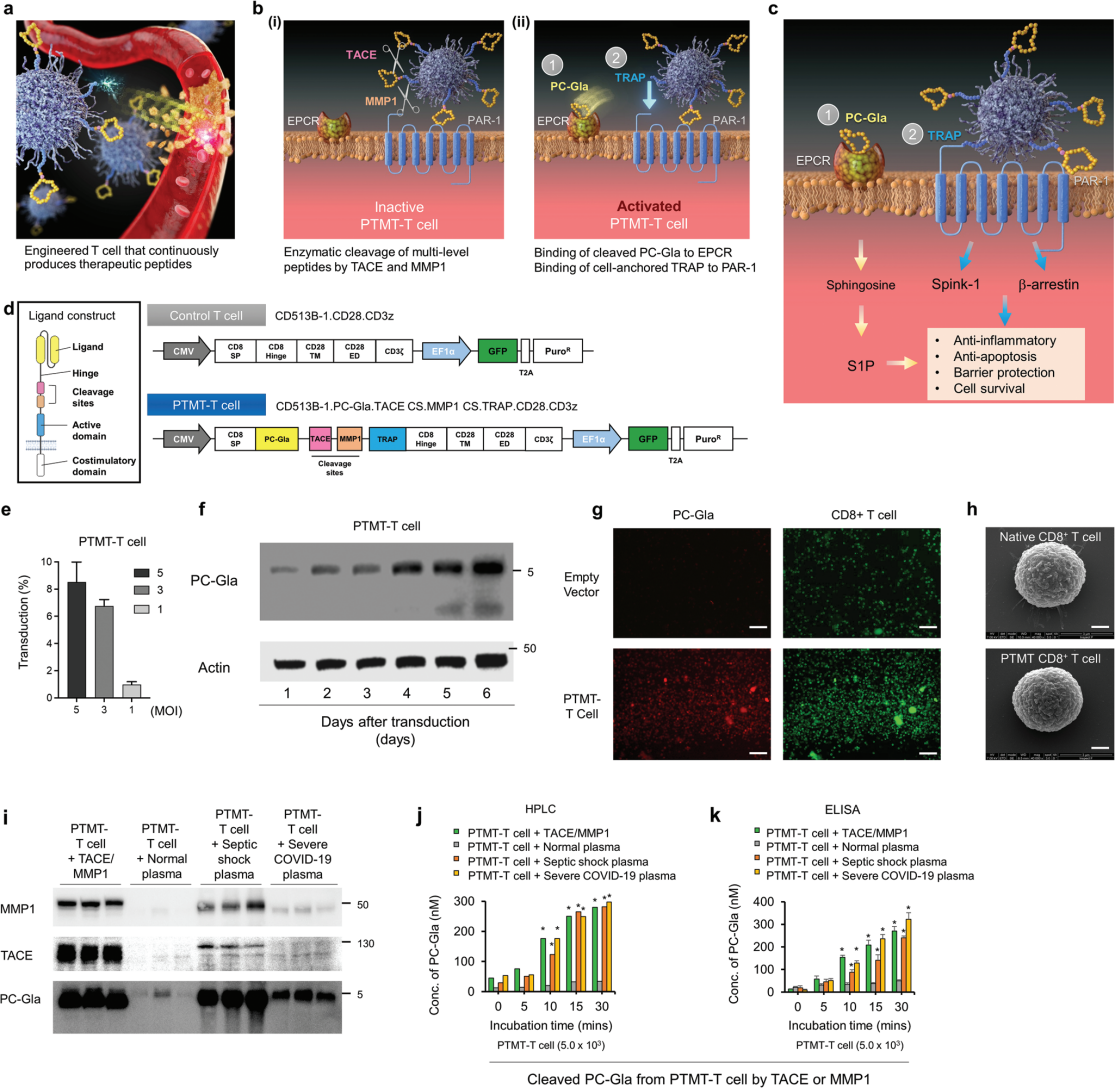
Design of therapeutic peptide‐producing T cell and its validation. a–c) Schematic illustration of mode of action of PC–Gla–TACE–MMP–TRAP (PTMT) T cell. During the blood circulation, serum‐abundant TACE and MMP1 cleave the peptide sequence, and the PC‐Gla and TRAP peptides are released. The PC‐Gla and TRAP bind to EPCR and PAR‐1, respectively, and, in turn, induce therapeutic effects. d) Vector design for PTMT. The therapeutic domains of PC‐Gla and TRAP, and TACE‐ and MMP1‐responsive cleavage sites are inserted. e) Transduction efficiency of PTMT‐T cell depending on the MOI. f) Western blot analysis of PTMT‐T cell‐secreted PC‐Gla depending on the culture time in vitro. g) Immunofluorescence images of PC‐Gla expressed in human CD8^+^ PTMT‐T cells. Scale bar, 75 µm. h) Scanning electron microscopy images of native and PTMT CD8^+^ T cells. Scale bar, 1 µm. i) Western blot analysis of MMP1, TACE, and PC‐Gla expressed in PTMT‐T cells when they were exposed to infectious disease patients’ plasma (*n* = 3/each group). The infectious diseases include septic shock and COVID‐19. j) High‐performance liquid chromatography (HPLC) analysis of gPC‐Gla expressed in PTMT‐T cells upon the exposure to infectious disease patients’ plasma. k) Enzyme‐linked immunosorbent assay (ELISA) analysis of PC‐Gla expressed in PTMT‐T cells upon the exposure to infectious disease patients’ plasma. Statistics, significance: The experiment was performed at least three times with replicates. Data are presented as mean ± SEM. *P*‐values are calculated using an ANOVA (j,k). *p < 0.05, ^**^ *p* < 0.01.

We engineered human T cells to continuously produce therapeutic peptide, and thus, target vascular and tissue inflammation (**Figure** **1**a). In the PTMT‐T cell, the MMP1‐ and TACE‐responsive cleavage sites were introduced to prevent therapeutic peptides from enzymatic degradation by the MMP and TACE during body circulation (Figure 1b). The therapeutic peptides expressed at the surface of T cell possess multi‐level structures, including the therapeutic moieties of *γ*‐carboxyglutamic acid of protein C (PC‐Gla) and thrombin receptor agonist peptide (TRAP), and the cleavage site responsive for matrix metallopeptidases 1 (MMP1) and tumor necrosis factor alpha converting enzyme (TACE), respectively (Figure 1b‐i). When the peptide‐expressing T cell is administered, TACE and MMP1 interact with the cleavage sites. The released PC‐Gla binds to EPCR, which is exposed on the cell membrane, and the cell‐bound TRAP binds to transmembrane protein PAR‐1 (Figure 1b‐ii). Such peptide–receptor and peptide–transmembrane protein interactions ultimately lead to therapeutic effects by suppressing inflammation and apoptosis (Figure 1c). These engineered therapeutic peptide‐expressing chimeric T cells, termed PC–Gla–TACE–MMP–TRAP–T cells (PTMT–T cells) (Figure 1d). Further, a multiplicity of infection (MOI)‐dependent transduction efficiency upon lentiviral vector treatment was analyzed in PTMT‐T cells and control T cells, respectively (Figure 1e; Figure S1, Supporting Information).

### 2.2 Validation of PC‐Gla Expression in the PTMT‐T Cell

We validated the expression of PC‐Gla in the PTMT‐T cell with western blot analysis and found an increase of the expression level with time (Figure 1f). The analysis by enzyme‐linked immunosorbent assay (ELISA) also demonstrated the time‐ and cell number‐dependent expression of PC‐Gla in the PTMT‐T cell (Figure S2, Supporting Information). As the concentration of PC‐Gla increased over time and with PTMT‐T cell proliferation, expression of peptide on the surface of the T cell was considered to be maintained regardless of cell replication. The matrix‐assisted laser desorption/ionization time‐of‐flight (MALDI‐TOF) confirmed the molecular weight of the PTMT‐T cell expressed PC‐Gla to be identical with that of PC‐Gla standard (Figure S3, Supporting Information). The immunofluorescence validation also confirmed that the PTMT‐T cell expressed PC‐Gla at the cell membrane (Figure 1g). The scanning electron microscopy (SEM) images showed no notable morphological difference of PTMT‐T cell after the transduction (Figure 1h). We verified whether the PC‐Gla was released from multi‐level peptides in the presence of MMP1 and TACE using immunoblotting (Figure 1i). When we treated PTMT‐T cell with MMP1 and TACE, the released PC‐Gla was confirmed. Furthermore, when the PTMT‐T cell was exposed to patients’ plasma of septic shock and severe COVID‐19, PC‐Gla was observed (Figure 1i), suggesting that the PC‐Gla was released in response to the infectious disease conditions. We validated the release of PC‐Gla from the PTMT‐T cell upon the exposure to septic shock and COVID‐19 patients’ plasma using high‐performance liquid chromatography (HPLC) (Figure 1j) and ELISA (Figure 1k). According to the results, the concentration of cleaved PC‐Gla from PTMT‐T cell exceeded 200 nm after 15 min of incubation, and then, increased continuously up to 300 nm after 30 min of incubation, which indicates physiological anti‐septic effect.^[^ ^18^ ^]^ The PTMT‐T cells secreted a low level of inflammatory cytokines for immune activation (Figure S4, Supporting Information). Furthermore, to be used as a therapeutic platform, a supplement for cell delivery and preservation is needed. The PTMT‐T cells showed enhanced viability when RGX365, a protopanaxatriol type ginsenoside fraction, was added as a supplement (Figure S5, Supporting Information).

### 2.3 PTMT‐T Cells Alternated EPCR Shedding by MMP1

**Figure 2:**
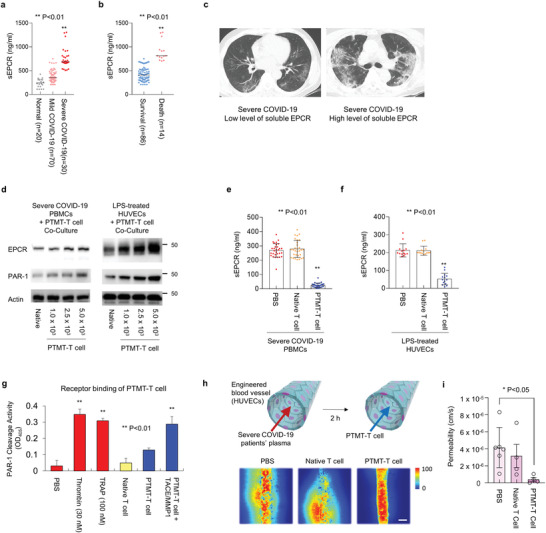
PTMT‐T cell protects endothelial protein C receptor (EPCR) of an infected blood vessel. a) The expression level of soluble EPCR (sEPCR) depending on the severity of COVID‐19 infectious diseases. (normal [*n* = 20], mild COVID‐19 [n = 70], and severe COVID‐19 [n = 30]). b) The expression level of sEPCR in survived and deceased individuals. c) The computed tomography images of COVID‐19 patients’ lung tissues. The degree of inflammation is related to the expression level of sEPCR. Representative images from each group are shown (*n* = 5). d) Western blot analysis of EPCR and PAR‐1 in severe COVID‐19 patients’ peripheral blood mononuclear cells (PBMCs) and human umbilical vein endothelial cells (HUVECs) when cocultured with PTMT‐T‐ cells. As the number of PTMT‐T cell increases, the *expression* levels of EPCR and PAR‐1 increase. e) Concentration of sEPCR cleaved by severe COVID‐19 patients’ PBMCs when cocultured with PTMT‐T cells. f) Concentration of sEPCR cleaved by HUVECs when cocultured with PTMT‐T cells. g) PAR‐1 cleavage activity (**p < 0.01). h) Recovery of the damaged engineered blood vessel by the PTMT‐T cells. The engineered blood vessel, which was formed in organ‐on‐a‐chip platform was damaged by the severe COVID‐19 patients’ plasma, and recovered after the administration of PTMT‐T cell (24 h coculture). Scale bar, 200 µm. i) Quantification of transendothelial permeability (*n* = 6/each group, *p < 0.05). Statistics, significance: The experiment was performed at least three times with replicates. Data are presented as mean ± SEM. *P*‐values are calculated using an ANOVA (a,b,e,f,g,i). *p < 0.05, ^**^ *p* < 0.01 (PBS (*n* = 6) and native and PTMT‐T cell (*n* = 4).

From the ELISA assay of severe COVID‐19 patients’ plasma, we found that the concentration of soluble endothelial protein C receptor (sEPCR) was increased as the severity of COVID‐19 increased (**Figure** **2**a). The concentration of sEPCR was further increased in the deceased individuals compared to survived cases (Figure 2b). The computed tomography (CT) images also support the plasma assay results, showing a higher inflammation in lung tissue in the case of the high level of sEPCR (Figure 2c). These clinical signatures commonly indicate that the level of sEPCR in COVID‐19 patients is highly correlated with clinical outcomes. The increased level of sEPCR originates from blood vessel damage. In the severe inflammatory stage after the infection, the EPCR bound in the membrane is damaged and becomes a soluble form due to the delamination from the membrane.^[^ ^19^ ^]^ Here, we found that the PTMT‐T cells can prevent the damage of membrane‐bound EPCR in neutrophil‐included PBMCs and human umbilical vein endothelial cell (HUVEC). For this study, the PTMT‐T cells were cocultured in the absence of direct contact with the severe COVID‐19 patients’ PBMCs (i.e., paracrine‐mediated interaction), and the expression level of EPCR in severe COVID‐19 patients’ PBMCs was analyzed with western blot. The expression level of PAR‐1 was also analyzed to confirm the effectiveness of PTMT‐T cells for the protection of transmembrane proteins responsible for the inflammation. The expression levels of EPCR and PAR‐1 were increased as the number of PTMT‐T cells increased (Figure 2d). The PTMT‐T cell also protected the EPCR and PAR‐1 in lipopolysaccharide (LPS)‐stimulated HUVECs. In turn, the levels of sEPCR in severe COVID‐19 patients’ PBMC culture media (Figure 2e) and LPS‐treated HUVECs media (Figure 2f) were decreased when the PTMT‐T cells were cocultured. We also measured the ability of thrombin, TRAP, and PTMT‐T cell to activate PAR‐1. The data show that TRAP of PTMT‐T cell is capable of activating PAR‐1 of endothelial cells in the presence of TACE and MMP1 (Figure 2g). The TRAP of PTMT‐T cell is capable of activating PAR‐1 of endothelial cells in the presence of TACE and MMP1. The effectiveness of the PTMT‐T cell was demonstrated using the blood vessel‐on‐a‐chip platform, which was fabricated using HUVECs. The engineered blood vessel was disrupted for 2 h by introducing severe COVID‐19 patients’ plasma (Figure 2h) or tumor necrosis factor (TNF)‐*α* (Figure S6, Supporting Information). For the comparison of barrier function, the fluorescence images were color‐mapped with custom‐written MATLAB codes and the color was displayed in an arbitrary unit. As shown in fluorescence intensity‐mapped images, the PTMT‐T cell recovered the barrier function of the engineered blood vessel (Figure 2g). These results indicate that the PTMT‐T cells protect the EPCR and PAR‐1 in the PBMCs and HUVECs and help the recovery of the vascular integrity in vitro.

### 2.4 PTMT‐T Cells Suppress Cytokine Release Syndrome in Severe COVID‐19 Patients’ PBMCs and LPS‐Treated HUVECs

**Figure 3:**
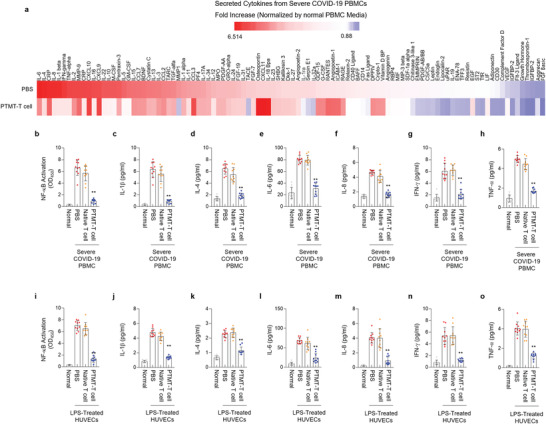
The PTMT‐T cell suppresses the cytokine storm in severe COVID‐19 patients’ PBMCs and HUVECs. a) Cytokine array results of severe COVID‐19 patients’ PBMCs. The coculture of PBMCs with PTMT‐T cell reduced the expression levels of inflammatory cytokines (*n* = 3/each group). b–h) ELISA results of NF‐*κ*B activity and released cytokines from severe COVID‐19 patients’ PBMCs (*n* = 10/each group, **p < 0.01). b) NF‐*κ*B activity. Expression levels of c) Interleukin (IL)‐1*β*, d) IL‐4, e) IL‐6, f) IL‐8, g) interferon (IFN)‐*γ*, and h) tumor necrosis factor (TNF)‐*α*. i–o), ELISA results of NF‐*κ*B activity and released cytokines from lipopolysaccharide (LPS)‐treated HUVECs (*n* = 10/each group, **p < 0.01). NF‐*κ*B activity (i). Expression levels of IL‐1*β* (j), IL‐4 (k), IL‐6 (l), IL‐8 (m), IFN‐*γ* (n), and TNF‐*α* (o). Statistics, significance: The experiment was performed at least three times with replicates. Data are presented as mean ± SEM. *P*‐values are calculated using an ANOVA (b–o). *p < 0.05, ^**^ *p* < 0.01.

The capability of PTMT‐T cell for the suppression of cytokine release syndrome was evaluated by cytokine array. The severe COVID‐19 patients’ PBMCs were cocultured with PTMT‐T cells without direct contact (i.e., paracrine‐mediated interaction) and quantified the fold change of inflammatory cytokines in culture media. The cytokine array showed that the PTMT‐T cells reduced the cytokine secretion related to the cytokine storm, including the IL‐4, IL‐8, IL‐1*β*, and TNF‐*α* (**Figure** **3**a). The ELISA assay also exhibited similar results, downregulated NF‐*κ*B activity (Figure 3b), and reduced inflammatory cytokines such as IL‐1*β*, IL‐4, IL‐6, IL‐8, IFN‐*γ*, and TNF‐*α* (Figure 3c–h). The LPS‐treated HUVECs displayed suppressed inflammatory signaling NF‐*κ*B (Figure 3i) and inflammatory cytokine secretion (Figure 3i–o). These results clearly demonstrate that the PTMT‐T cell is capable of downregulating inflammation‐related signaling pathway NF‐*κ*B and of suppressing cytokine storm in severe COVID‐19 patients’ PBMCs and LPS‐treated HUVECs.

### 2.5 PTMT‐T Cells Rescue the Survival Rate in Animal Model and Ameliorate Tissue Damage

**Figure 4:**
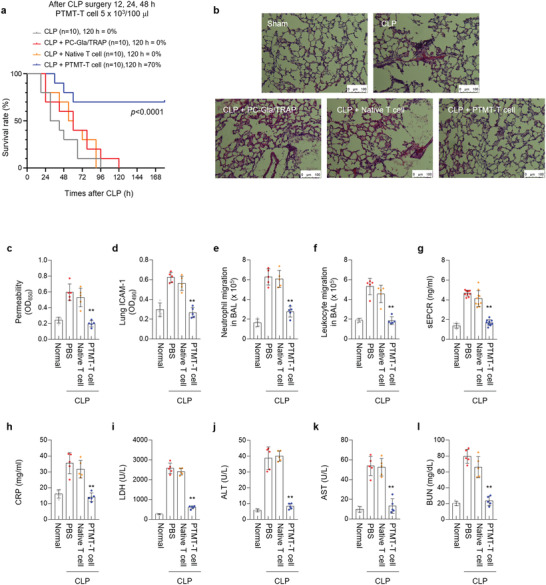
PTMT‐T cell suppresses the cytokine storm and lung tissue damage in septic mice models. a) Survival rate of CLP‐operated mice after the intravenous injection of PTMT‐T cells. The PTMT‐T cell rescued the survival rate of septic mice models by 70% (*n* = 10/each group). b) Hematoxylin and Eosin (H&E) staining of CLP‐operated mice’ lung tissue. The administration of PTMT‐T cell reduced the lung organ damage. Representative images from each group are shown (*n* = 5). Scale bar, 100 µm. c) Vascular permeability of septic mice. d) Expression level of lung intercellular adhesion molecule (ICAM)‐1. e) Neutrophil migration in bronchoalveolar lavage (BAL). f) Leukocyte migration in BAL. g) Concentration of sEPCR. h–l) Concentration of tissue damage markers. C‐reactive protein (CRP) (h). Lactate dehydrogenase (LDH) (i). Alanine aminotransferase (ALT) (j). Aspartate aminotransferase (AST) (k). Blood Urea Nitrogen (BUN) (l). (*n* = 5/each group, **p < 0.01). Statistics, significance: The experiment was performed at least three times with replicates. Data are presented as mean ± SEM. *P*‐values are calculated using an ANOVA (c–l). *p < 0.05, ^**^ *p* < 0.01.

For in vivo validation, we adapted the cecal ligation and puncture (CLP) mice model.^[^ ^20^ ^]^ The CLP model is a most widely used sepsis model and displays clinically‐relevant signature with COVID‐19 and sepsis, including the cytokine storm, severe inflammation, and multiple‐organ failure.^[^ ^21^ ^]^ The CLP‐operation induced the death of mice models within 96 h. Although the direct administration of PC‐Gla and TRAP through the intravenous injection could not rescue the survival rate, the PTMT‐T cells rescued the survival rate by 70% (**Figure** **4**a; Figure S7 and S8, Supporting Information). The hematoxylin and eosin (H&E) staining of CLP‐operated septic mice models’ lung tissue showed that the PTMT‐T cells prevented vascular damage and lung tissue inflammation (Figure 4b). However, the direct administration of PC‐Gla and TRAP could not inhibit lung tissue damage. The administration of PTMT‐T cells restored the pathological features into the normal range, including the vascular integrity disruption (Figure 4c), expression of lung intercellular adhesion molecule (ICAM)‐1 (Figure 4d), and neutrophil (Figure 4e) and leukocyte migration in bronchoalveolar lavage (BAL) (Figure 4f). The concentration of sEPCR was similar to that of the normal case, suggesting the protection of EPRC by PTMT‐T cells (Figure 4g). The tissue damage markers including the C‐reactive protein (CRP), lactate dehydrogenase (LDH), alanine aminotransferase (ALT), aspartate aminotransferase (AST), and blood urea nitrogen (BUN) (Figure 4h–l) were also maintained at a low level, which indicates recovery of liver and kidney.^[^ ^22^ ^]^ The cytokine storm was suppressed in the CLP mice models upon administration of the PTMT‐T cells, as confirmed by the reduced IL‐1*β*, IL‐6, IL‐8, and TNF‐*α* (Figure S9, Supporting Information). The administration of PTMT‐T cells in CLP‐operated mouse model regulated the neutrophil and leukocyte number, which indicated the recovery and activation of immunity (Figure S10, Supporting Information).^[^ ^23^ ^]^ The in vivo results collectively indicate that the PTMT‐T cell is an effective therapeutics in the suppression of cytokine storm and tissue damages.

## 3 Discussion

Over the past decade, peptide‐based therapeutics have undergone a resurgence of interest, as pharmaceutical companies have come to acknowledge that they are a good alternative to small molecules for addressing unmet medical needs.^[^ ^24^ ^]^ However, extension of the half‐life is one of the key challenges to overcome in the field of peptide‐based therapeutics.^[^ ^25^ ^]^ Despite the growing interest in therapeutic peptides, their clinical application is frequently compromised due to the hampered capability in vivo.^[^ ^26^ ^]^ Such limited efficacy of peptide drugs largely originates from the loss of functionality during the body circulation.

Here, we report the effects of PTMT‐T cells on resolving inflammatory responses and reducing tissue damage. As shown in this study, the PC‐Gla and TRAP showed a reasonable effect in the in vitro‐based analysis (Figure 2h). To validate the efficacy in vivo, we adapted CLP mice model as it is widely used for severe infectious disease studies, such as sepsis, and can reflect various symptoms observed in severe infectious diseases.^[^ ^27^ ^]^ In severe COVID‐19 patients, symptoms such as septic shock and acute respiratory distress syndrome are commonly observed, which are the leading cause of death.^[^ ^28^ ^]^ In the CLP‐operated mice model, PC‐Gla and TRAP showed no or minimal effect when they were administered through the intravenous injection (Figure 4). To resolve the current unmet issue of peptide‐based therapeutics, we adapted T cell technology not only as a carrier but also as a manufacturing vehicle. The engineered T cells stably expressed PC‐Gla and TRAP therapeutic peptides on the cell surface. The therapeutic effects were increased because the PTMT‐T cells continuously express on the membrane and can maintain the peptides through the lifetime of the cells (Figure 1d–I; Figure S7, Supporting Information). It has been previously reported that inflammatory responses lead to MMP production.^[^ ^18^ ^]^ Accordingly, the chimeric T cells were designed with MMP and TACE‐responsive cleavage sites to release free form of PC‐Gla, and thereby provide a higher degree of anti‐inflammatory activity than in the non‐cleavable form. In addition, by leaving TRAP attached on the cell surface, it could act more efficiently at the inflammatory sites. In particular, intravenous injection of PTMT‐T cells alleviated the systemic inflammation and attenuated mortality in a septic mouse model, resulting in a 70% survival rate for the CLP‐operated septic mice for more than 168 h post‐induction (Figure 4a), demonstrating that the mice had fully recovered. For these reasons, the efficacy was significantly enhanced compared to the direct administration of peptide therapeutics at a single shot. Furthermore, the incorporation of cleavable sites in the multi‐level peptide on the cell surface allows evasion from the enzymatic digestion, which is inevitable in the in vivo condition. In addition, we incorporated CD28 costimulatory domain with CD3 activating cytoplasmic domains to add the benefit of conferring on the engineered T cells some immune activation with the potential to secrete cytokine to the surrounding microenvironment. This is due to the reduction in the T cell population upon infection; hence, it is essential to supplement the stimulated T cells in the infected environment (Figure 1b).^[^ ^29^ ^]^ To the best of the authors’ knowledge, the concept of engineered peptide‐expressing chimeric T cell has not yet been reported. This concept is different from the chimeric antigen receptor‐T (CAR‐T) technology as the PTMT‐T cell is considered to lead the release of cytokine in a low level, avoiding degranulation and release of lytic granules. The purpose of PTMT‐T cell is not to eliminate the target cells but to protect normal cells by suppressing hypercytokinemia, vascular damage, and lung tissue failure. Therefore, it is considered that the effects of CAR‐T cells inducing direct cell death would not occur in PTMT‐T cell.

The engineered chimeric T cell therapy demonstrates promising effects for the treatment of COVID‐19 and sepsis in vitro and in vivo. The results indicate that administration of the PTMT‐T cells can suppress the cytokine storm in parallel with the downregulation of NF‐*κ*B signaling, and the recovery of vascular damage, as shown in the blood vessel‐on‐a‐chip model and LPS‐treated HUVEC model. In the mice model, the rescue of survival rate, suppression of cytokine storm, and prevention of tissue damage was observed.

Since CD4^+^ and CD8^+^ T cells recognize the N protein of SARS‐CoV‐2 and activate adaptive immune, we intended to use PTMT‐T cell treatment strategy to supplement healthy T cells and recover the T cell number. Previous observation of severe COVID‐19 patients’ blood reported a reduced number of lymphocytes, especially T cells.^[^ ^30^ ^]^ Furthermore, the exhaustion of CD8^+^ T cell in the COVID‐19 patients was also observed.^[^ ^31^ ^]^ As engineered chimeric T cell technology relies on infusion of T cells, this can lead to immune activation and the reduced T cell can be replenished. In addition, the utilization of patient‐derived T cell can avoid the immune rejection issue. In this study, CD8^+^ T cell was selected as a target cell to effectively treat COVID‐19 and improve the related symptoms; however, this technology is not limited to specific types of cells and has the potential to be applied to more diverse cells.

## 4 Conclusion

In the present study, we demonstrated engineered therapeutic peptide‐expressing chimeric T cell as a therapeutic modality for treatment of severe infectious diseases. A new peptide‐based therapeutic system was designed bearing two functional peptides, PC‐Gla and TRAP; and cleavages sites, TACE and MMP‐1, allowing them to work together on the cell surface at the inflammation site to attenuate disease‐related inflammatory responses. Our findings reveal that immune cell‐based peptide‐delivery method could be exploited to resolve critical issues of peptide‐based therapeutics, such as instability in the in vivo environment, limited effective time with a single administration, and single targeting with use of a single peptide drug. This engineered T cell technique can be further extended to treat other types of diseases by designing appropriate peptides on the cell surface or secretion to the extracellular matrix and environment. We envision that the peptide‐expressing chimeric T cell technology may shed light on the treatment of incurable chronic diseases. It should be noted that because the CLP‐induced mice model was used, the efficacy of PTMT‐T cell therapy needs to be further validated in an appropriate in vivo setting using a virus infection‐induced animal model before moving forward to clinical trials on patients with severe infectious diseases, such as COVID‐19.

## 5 Experimental Section

### Preparation of Therapeutic Peptide Vector

Therapeutic peptide (PTMT, PC–Gla–TACE–MMP–TRAP), transmembrane (CD28 TM), co‐stimulatory (CD28 ED), and activating (CD3*ζ*) cytoplasmic domains were prepared using DNA oligo synthesis (Cosmo Genetech, Seoul, Korea). The CD28 TM, CD28 ED, and CD3*ζ* construct were cloned in pCDH‐CMV double promoter lentiviral vector (Addgene). Then, PTMT gene was inserted between signaling (CD8 SP) and hinge (CD8 Hinge) domains.

### Lentiviral Vector Supernatant Production, Titration, and Transduction

Lentiviral vector supernatants were generated by co‐transfecting 1 mg of lentiviral transfer vector (CD513B‐1.CD28.CD3z for control T cell and CD513B‐1.PC‐Gla.TACE CS.MMP1 CS.TRAP.CD28.CD3z for PTMT‐T cell, respectively), 0.5 mg of Gag/Pol, 0.25 mg of REV, and 0.25 mg of vesicular stomatitis virus (VSV‐G) packaging plasmids into Lenti‐X 293T cells (Takara Bio, Japan) in a 12‐well plate using linear PEI reagent. Supernatants were collected at 48 h after transfection and filtered through a 0.45‐mm polyvinylidene fluoride (PVDF) syringe filter (Corning Life Sciences, Tewksbury, MA, USA). To determine the titer, HT1090 cells were infected in a 24‐well plate with serial dilutions of the lentiviral vector supernatants in the presence of polybrene (8 mg mL^−1^) (Sigma–Aldrich, St. Louis, MO, USA) and analyzed 3 days post‐transduction by flow cytometry analysis using the BD FACS Fortessa (BD Biosciences, San Jose, CA, USA). Transductions of T cells were performed on 2 × 10^4^ cells per well in a 24‐well plate followed by flow cytometric analysis of GFP expression 3 days post‐transduction.

### Cell Culture

Human T cells were isolated using Pan T cell isolation kit (Miltenyi Biotec, Bergisch Gladbach, Germany) and mouse T cells were isolated using T cell isolation kit (Miltenyi Biotec, Bergisch Gladbach, Germany), respectively. Both human and mouse T cells were maintained in RPMI medium supplemented with 10% fetal calf serum, 1% penicillin–streptomycin, and 100 U IL‐2.

### Plasma Sample

Whole blood was collected from patients admitted at the Yeungnam University Medical Center after they were diagnosed with the SARS‐CoV‐2 infection at the Public Health Center in Daegu, Republic of Korea. Patients with COVID‐19 sepsis were defined using criteria provided by the Sepsis Consensus Conference Committee.^[^ ^32^ ^]^ Among the patients admitted at the Yeungnam University Medical Center, pneumonia and septic shock patients were collected. Healthy volunteers were used as controls. Clinical data were collected for all the patients. Plasma samples were prepared by centrifugation at 2000 × *g* for 5 min within 12 h after whole blood collection. The human study protocol was approved by the Institutional Review Board of Yeungnam University Hospital at Daegu in Korea (YUH 2020‐03‐057, 2020‐05‐031‐001). These experiments were carried out with the full, informed consent of the subjects. Whole blood was collected from patients after approval for use in the study. And the analysis of blood samples received from the hospital was conducted in blind. Viruses in the blood were observed in COVID‐19 patients, and progression toward severe respiratory disease or sepsis was progressed based on the cohort study of patients. Patients with COVID‐19 sepsis were defined using criteria provided by the Sepsis Consensus Conference Committee.^[^ ^32^ ^]^ Eligible patients met the following criteria: age, over 18 years; and an acute onset medical condition with at least one of the following criteria: fever (tympanic temperature ≥ 38 °C at the nurse triage), suspected systemic infection, two or more systemic inflammatory response syndrome SIRS criteria, hypotension (systolic blood pressure < 90 mmHg), and/or shock. Healthy volunteers were used as controls. Clinical data were collected for all the patients. COVID‐19 patients were categorized depending on the disease severity. “Mild” is defined as patients receiving quarantine treatment in general ward or those with asymptomatic symptoms. “Severe” is defined as patients with ARDS, sepsis, or receiving intensive care in the ICU. It also includes patients treated with oxygen therapy or those that rely on mechanical breathing machines (i.e., ventilators). Similar to previous finding, it was noticeable that most severe patients were elderly with age over 70.^[^ ^30^ ^]^

### PBMC Isolation and Culture

Samples from healthy, SARS‐CoV‐2 pneumonia patients, or discharged patients were obtained from the Yeungnam University Medical Center. The relevant local Institutional Review Boards and Ethics Committees approved the study. Heparinized blood samples were used fresh within 4 h, and peripheral blood mononuclear cells (PBMCs) were separated from the blood using Ficoll–Hypaquek or NycoPrepk according to the manufacturer's recommendations. Following this, more refined PBMCs were obtained via MACSprep PBMC Isolation Kit and cultured in RPMI‐1640 with 1 mm sodium pyruvate, 2 mm L‐glutamine, 4.5 mg L^−1^ glucose, 10 mm HEPES, and 2 mg L^−1^ sodium bicarbonate.

### Human Soluble EPCR ELISA

EPCR immunoassay kits were obtained from R&D Systems. sEPCR level in citrated plasma was determined using human ELISA kits (Quantikine ELISA, R&D Systems, Minneapolis, MN, USA) according to the manufacturer's instructions.

### Western Blotting

GLA (Gamma‐Carboxyglutamyl) domain was detected by immunoblotting in PTMT‐T cell. The level of secreted GLA domain was evaluated in human MMP‐1 (Sigma, SPR3117)/TACE (Sigma, SRP6178)‐treated or severe COVID‐19 plasma‐treated PTMT‐T cell. EPCR and PAR‐1 were analyzed by immunoblotting, in PTMT‐T cell co‐incubated with separated PBMCs from severe COVID‐19 or LPS‐activated HUVECs. After sodium dodecyl sulfate polyacrylamide gel electrophoresis, the authors performed an immunoblotting assay with monoclonal mouse GLA (Gamma‐Carboxyglutamyl) domain antibody (ABIN124983, antibodies‐online GmbH), rabbit polyclonal to EPCR/CD201 antibody (ab151403, Abcam, United Kingdom), and monoclonal mouse PAR‐1 antibody (sc‐13503, Santa Cruz, USA).

### Characterization of PTMT‐T Cell

HPLC analysis of PC‐Gla was carried out with a Dionex Ultimate 3000 HPLC system (Sunnyvale, CA, USA) consisting of a binary pump with an online vacuum degasser, an automated sample injector, thermostatically controlled column compartment, and fluorescence detector. Analysis was carried out on a Gemini C‐18 column (250 × 4.6 mm id, 5 µm, Phenomenex, Torrance, CA). The mobile phase consisted of deionized water containing 0.1% trifluoroacetic acid (TFA) (eluent A) and acetonitrile containing 0.1% TFA (eluent B). A linear gradient elution of 30–60% (v/v) eluent B was applied for 20 min. Flow rate was 1.0 mL min^−1^ and sample injection volume was 20 µL. The mass spectrum of each construct monomer was confirmed by matrix‐assisted laser desorption ionization time of flight (MALDI‐ToF). MALDI‐ToF MS was carried out using a Bruker Daltonics Microflex MALDI‐ToF mass spectrometer (Bremen, Germany) with a 337 nm nitrogen laser. Mass spectra were obtained in the linear and positive‐ion mode with an acceleration voltage of 20 kV. A saturated solution of *α*‐cyano‐4‐hydroxycinnamic acid (CHCA) or sinapic acid in 50% acetonitrile, containing a final concentration of 0.1% trifluoroacetic acid, was used as the matrix solution. A CHCA matrix was chosen for analysis of fragments after enzyme digestion or sinapic acid for intact proteins. The analyte‐matrix solution was prepared at a ratio of 1:2 (analyte:matrix, v/v). Each mixture was thoroughly mixed and 1 µL of the analyte–matrix solution was deposited onto the sample plate and dried by vacuum evaporation. For SEM imaging, cells were washed three times with DPBS and fixed with 0.1 m sodium cacodylate and 0.1 m sucrose for 30 min at 4 °C. After washing with distilled water for 5 min, cells were dehydrated by serial additions of 50%, 75%, 90%, and 100% ethanol solutions for 5 min each. Then, cells were immersed in hexamethyl disilazane (HMDS) for 15 min at room temperature inside the hood. After drying the samples, the substrates were sputter‐coated with Pt to the thickness of 20 nm prior to measurements. SEM images were obtained using a HITACHI S‐4800 microscope (Hitachi, Japan).

### PAR‐1 Cleavage Assay

HUVECs at 90% confluence in 24‐well plates were transiently transfected with pRc/RSV containing ALP–PAR–1–TF–cDNA in antibiotic‐free Opti‐MEM medium using Lipofectamine (Invitrogen, USA) according to the manufacturer's instruction. On the following day, cells were washed and incubated in serum‐free medium for 5 h. Cells were then incubated for an additional hour with thrombin, TRAP, TFG, or TFMG. Conditioned medium was collected and centrifuged to remove cellular debris. Supernatant was collected, and ALP (alkaline phosphatase) activity was measured using EnzoLyteTM p‐nitrophenyl phosphate alkaline phosphatase assay kit (AnaSpec, San Jose, CA, USA) according to the manufacturer's instructions.

### Engineered Blood Vessel Experiment

The fabrication of engineered blood vessels and the permeability assay were performed as previously described.^[^ ^33^ ^]^ In brief, the engineered blood vessel was fabricated by using microneedles as templates. The collagen microchannels were fabricated by inserting in the sol‐state type I rat tail collagen (Corning, Bedford, MA, USA) and subsequently removing them after the gelation. The human umbilical vein endothelial cells were seeded in the luminal surface of the collagen channel and cultured for 5 days to make a tight vascular structure. The permeability assay was performed by introducing 40 kDa FITC‐Dextran solution (10 µm, Sigma–Aldrich, Saint Louis, MO, USA) into the perfusable blood vessel and monitoring the molecular transport using a confocal microscope (LSM700, Zeiss, Jena, Germany). The permeability assay was performed by introducing 40 kDa FITC‐Dextran solution (10 µm, Sigma–Aldrich, Saint Louis, MO, USA) into the perfusable blood vessel and monitoring the molecular transport using a confocal microscope (LSM700, Zeiss, Jena, Germany). For the color mapping, the acquired images were color‐mapped with custom‐written MATLAB (MathWorks) codes and the fluorescence intensity was displayed in an arbitrary unit. The mean fluorescence intensity values across microchannels were also analyzed with custom‐written MATLAB (MathWorks) codes. To induce COVID‐19‐mediated inflammation in the engineered blood vessel, the severe COVID‐19 patients’ plasma was introduced in the blood vessel and incubated for 2 h. As the plasma of SARS‐CoV‐2 patients is easily coagulated, it was diluted in 1:2 ratio (plasma: media) before the injection to ensure sufficient fluidity.

### Cytokines Profiling

PBMCs isolated from patient blood were treated with PTMT‐T cell (5 × 10^3^) for 12 h. Plasma pools of patients with normal or severe COVID‐19 were processed as indicated in the Human XL Cytokine Array Kit (R&D Systems, MN, USA). Developed films were scanned and the obtained images were analyzed using ImageJ version 1.43.

### NF‐*κ*B Activity

Nuclear extracts were prepared and TransAM assays were performed as previously described.^[^ ^34^ ^]^ The activity of individual NF‐*κ*B subunits was determined via ELISA using NF‐*κ*B Family Transcription Factor Assay Kit (43296; Active Motif, Carlsbad, CA, USA). Briefly, nuclear extracts (2 µg) were placed into wells of NF‐*κ*B consensus oligonucleotide‐coated 96‐well plates. Plates were incubated with NF‐*κ*B primary antibody and then binding was detected using HRP‐conjugated secondary antibody included with the kit. For analysis, the optical density (OD) at 450 nm was measured using a Tecan Spark microplate reader (Tecan, Austria GmbH, Austria).

### Inflammatory Cytokines IL‐1*β*, IL‐4, IL‐6, IL‐8, IFN‐*γ*, and TNF‐*α* ELISAs

Serum levels of inflammatory cytokines IL‐1*β* and TNF‐*α* were determined in SARS‐CoV‐2‐infected patients using human ELISA kits (Quantikine ELISA, R&D Systems, Minneapolis, MN, USA) according to the manufacturer's instructions. PBMCs isolated from patient blood were treated with PTMT‐T cell (5 × 10^3^) for 12 h. The results were expressed as pg mL^−1^.

### WST‐1 Cell Proliferation Assay

10 µL per well of WST‐1 reagent was added in purified PBMC and incubated at 37 °C with 5% CO_2_. At indicated time points, measurements of absorbance were taken at 480 and 600 nm (background) on Tecan Spark microplate reader.

### Animals and Husbandry

Male C57BL/6 mice (6–7‐weeks‐old, weighing 18–20 g) were purchased from Orient Bio Co. (Sungnam, Kyungki‐Do, Republic of Korea) and used after a 12‐day acclimatization period. The animals were housed, 5 per polycarbonate cage, under controlled temperature (20–25 °C) and humidity (40–45%) under a 12:12 h light/dark cycle, fed a normal rodent pellet diet, and supplied with water ad libitum. All animals were treated in accordance with the Guidelines for the Care and Use of Laboratory Animals issued by Chungnam National University.

### Cecal Ligation and Puncture

The CLP‐operated septic mouse model was prepared as previously described.^[^ ^20^ ^]^ Briefly, a 2 cm midline incision was made to expose the cecum and adjoining intestine. The cecum was then ligated tightly using a 3.0‐silk suture 5.0 mm from the cecal tip, punctured with a 22‐gauge needle, and then gently squeezed to extrude feces from the perforation site. The cecum was then returned to the peritoneal cavity, and the laparotomy site was sutured using 4.0‐silk. For sham operations, the cecum of animals was surgically exposed, but not ligated or punctured, and then returned to the abdominal cavity. Male C57BL/6 mice underwent CLP and were then tail vein injected with PTMT‐T (1, 2.5 or 5 × 10^3^ cells) at 12, 24, and 48 h after CLP (*n* = 5). The mice were euthanized 72 h after CLP treatment.

### Hematoxylin and Eosin Staining

Male C57BL/6 mice underwent CLP and were administered PTMT‐T cell intravenously at 12 h after CLP (*n* = 5). Mice were euthanized 96 h after CLP. To analyze the phenotypic change of lung in mice, lung samples were removed from each mouse, washed three times in PBS (pH 7.4) to remove remaining blood, and fixed in 4% formaldehyde solution (Junsei, Tokyo, Japan) in PBS, pH 7.4 for 20 h at 4 °C. After fixation, the samples were dehydrated through ethanol series, embedded in paraffin, sectioned into 4 µm sections, and placed on a slide. The slides were deparaffinized in a 60 °C oven, rehydrated, and stained with hematoxylin (Sigma, Saint Louis, MO, USA). To remove over‐staining, the slides were quick‐dipped three times in 0.3% acid alcohol, and counterstained with eosin (Sigma, Saint Louis, MO, USA). They were then washed in ethanol series and xylene, and then cover‐slipped. Light microscopic analysis of lung specimens was performed by blinded observation to evaluate pulmonary architecture, tissue edema, and infiltration of the inflammatory cells.

### In Vivo Permeability and Leukocyte/Neutrophil Migration Assays

CLP‐operated mice were injected with PTMT‐T cell intravenously. After 12 h, 1% Evans blue dye solution in normal saline was injected intravenously into each mouse. Thirty minutes later, the mice were killed, and the peritoneal exudates were collected after being washed with normal saline (5 mL) and centrifuged at 200 × *g* for 10 min. The absorbance of the supernatant was read at 650 nm. The vascular permeability was expressed in terms of dye (*μ*g per mouse), which leaked into the peritoneal cavity according to a standard curve of Evans blue dye. For assessment of leukocyte/neutrophil migration, CLP operated mice were treated with PTMT‐T cell after CLP surgery 12 h. The mice were then sacrificed and the bronchoalveolar lavage (BAL) were washed with 0.8 mL of normal saline.^[^ ^35^ ^]^ BAL fluid (200 µL) was counted by auto hematology analyzer (Mindray, BC 5000 Vet) at the Chiral Material Core Facility Center of Sungkyunkwan University. The results were expressed as leukocyte/neutrophil × 10^5^ per BAL fluid.

### Expression of ICAMs

The expression of intercellular adhesion molecule‐1 (ICAM‐1) on lung tissues was determined by a direct ELISA. The lysed lung tissues were coated onto Nunc‐Immuno MicroWell 96 well plates and incubated overnight at 4 °C. After washing, anti‐mouse monoclonal ICAM‐1 antibodies (Millipore Corporation, Billerica, MA, USA, 1:50 each) were added. After 1 h (37 °C, 5% CO_2_), the cells were washed three times and then 1:2000 peroxidase‐conjugated anti‐mouse IgG antibody (100 *μ*L; Sigma, Saint Louis, MO, USA) was added for 1 h. The cells were washed again three times and developed using the o‐phenylenediamine substrate (Sigma, Saint Louis, MO). Colorimetric analysis was performed by measuring absorbance at 490 nm. All measurements were performed in triplicate wells.

### Clinical Chemistry and Cytokine Level in Septic Mice Plasma

Fresh serum was used for assaying aspartate transaminase (AST), alanine transaminase (ALT), blood urea nitrogen (BUN), C‐reactive protein (CRP), and LDH using biochemical kits (Mybiosource, San Diego, USA). To determine the concentrations of IL‐1*β*, IL‐6, IL‐8, and TNF‐*α*, commercially available ELISA kits were used according to the manufacturer's protocol (R&D Systems, MN, USA). Values were measured using an ELISA plate reader (Tecan, Austria GmbH, Austria).

### Statistical Analysis

All the in vitro and in vivo data were analyzed via two‐tailed unpaired *t*‐test using the GraphPad prism 8 software; the prepared sample sizes were *n* ≥ 3, and the statistical significance was set at *P* < 0.05. More detailed information for each experiment is provided in the Figure legend. All data normalization processes were carried out according to the manufacturer's protocol. Data transformation and evaluation of outliers were not used in the authors’ study.

## Conflict of Interest

The authors declare no conflict of interest.

## Author Contributions

*Conceived the project*: H.K., B.S., and E.U.S. *Provided patient samples*: J.H.A. *Analyzed data*: M.K., H.S., G.Y.S., E.J.P., D.H.N., and S.‐W.C. *Conceptualized, supervised the project, and wrote the manuscript*: H.N.K., H.H.P. and W.L.

## Supporting information

Supporting Information

## Data Availability

The data that support the findings of this study are available on request from the corresponding author. The data are not publicly available due to privacy or ethical restrictions.
