# Supplementary material for: Cleavage‐Responsive Biofactory T Cells Suppress Infectious Diseases‐Associated Hypercytokinemia
Source: Adv Sci (Weinh). 2022 Jun 25;9(26):2201883. doi: 10.1002/advs.202201883 (PMC9475519; doi:10.1002/advs.202201883)
Supplement: Supplementary file 1 — Supporting Information [file ADVS-9-2201883-s001.pdf]

ADVANCED SCIENCE
Open Access

Supporting Information

for *Adv. Sci.*, DOI 10.1002/advs.202201883

Cleavage-Responsive Biofactory T Cells Suppress Infectious Diseases-Associated Hypercytokinemia

Hyelim Kim, Boram Son, Eun U Seo, Miji Kwon, June Hong Ahn, Heungsoo Shin, Gyu Yong Song, Eun Ji Park, Dong Hee Na, Seung-Woo Cho, Hong Nam Kim, Hee Ho Park* and Wonhwa Lee**

Supporting Information

Cleavage-responsive biofactory T cells suppress infectious diseases-associated hypercytokinemia

Hyelim Kim, Boram Son, Eun U Seo, Miji Kwon, June Hong Ahn, Heungsoo Shin, Gyu Yong Song, Eun Ji Park, Dong Hee Na, Seung-Woo Cho, Hong Nam Kim, Hee Ho Park*, and Wonhwa Lee**

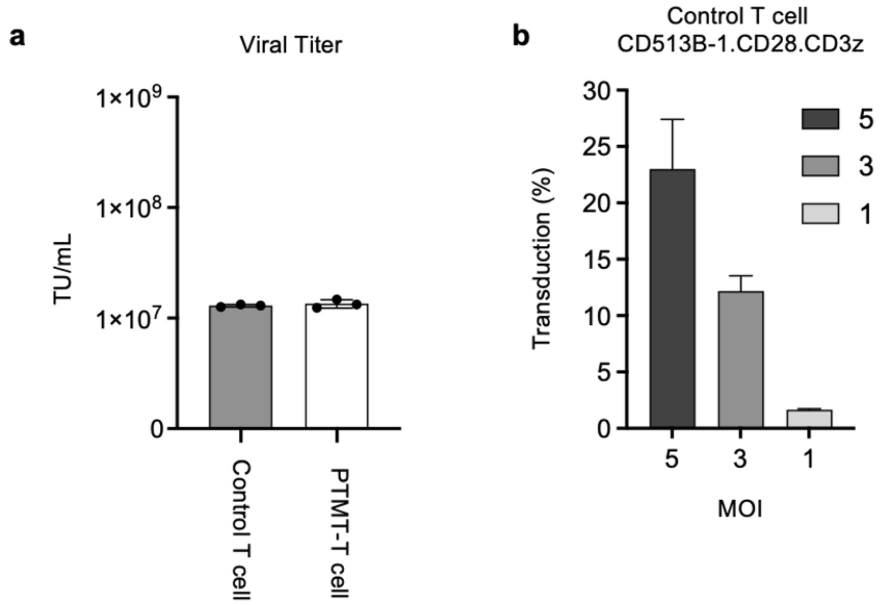

Figure S1. Viral titer and transduction efficiency of produced virus. (a) Viral titer. (b) Transduction efficiency of control T cell depending on the multiplicity of infection (MOI).

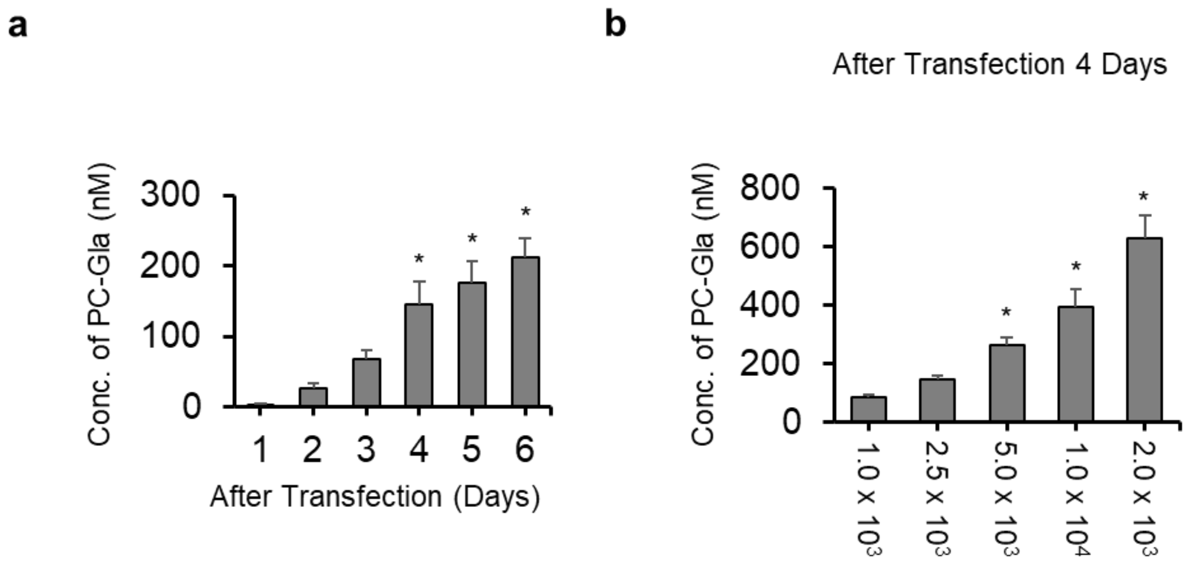

Figure S2. Time- and cell number-dependent concentration of PC-Gla. (a) Concentration of PC-Gla after the transduction. (b) Concentration of PC-Gla with respect to the number of PTMT-T cells. Statistics, significance: The experiment was performed at least three times with replicates. Data are presented as mean ± SEM. *P*-values are calculated using an ANOVA. $^{*}p < 0.05$.

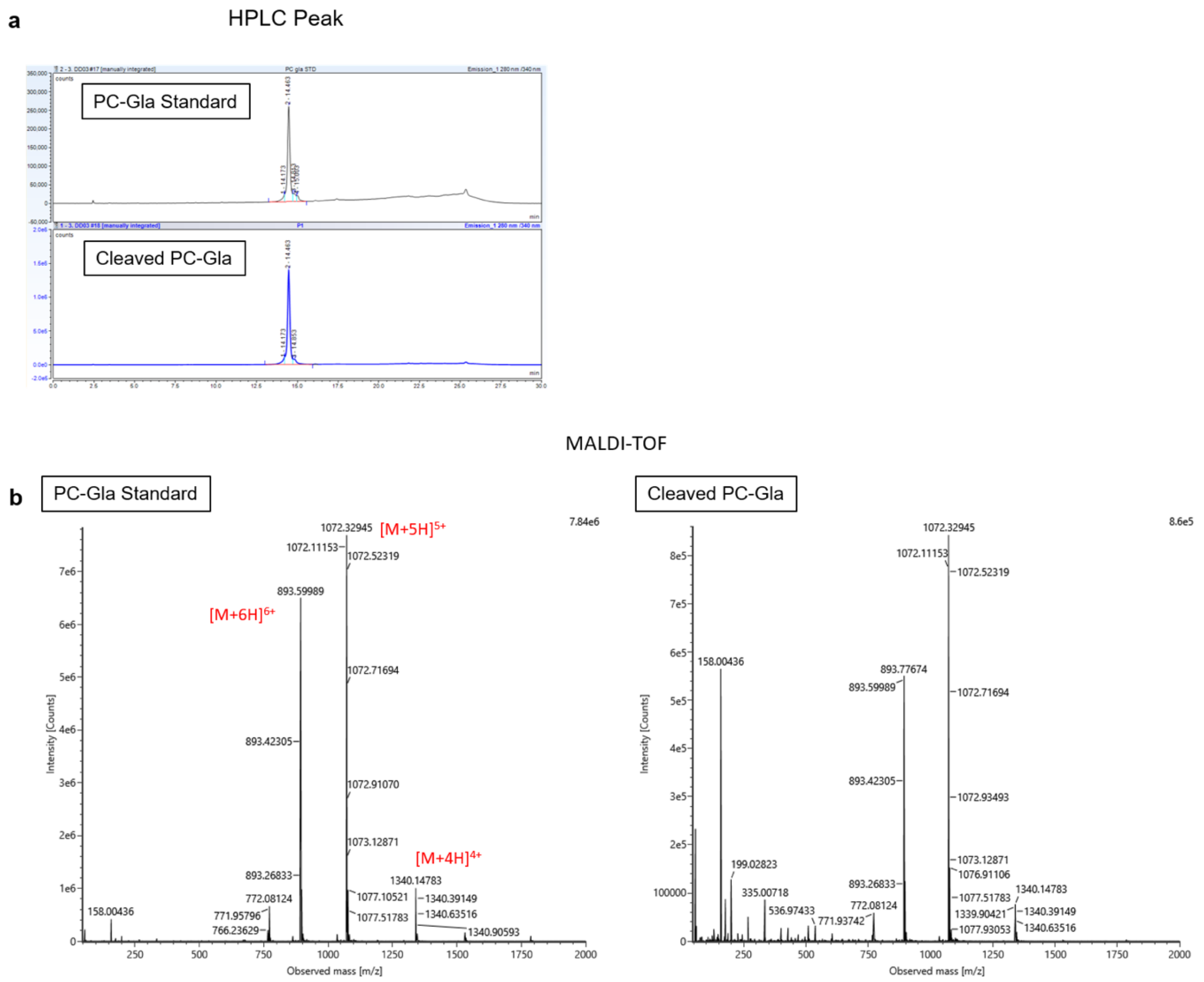

Figure S3. Analysis of cleaved PC-Gla. (a) High-performance liquid chromatography (HPLC) analysis of cleaved PC-Gla. (b) Matrix-assisted laser desorption/ionization time-of-flight (MALDI-TOF) analysis of cleaved PC-Gla. The experiment was performed at least three times with replicates.

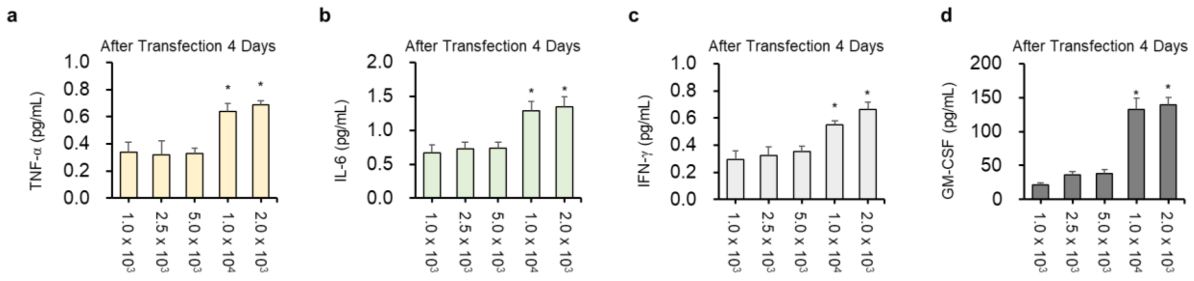

Figure S4. Secreted cytokines from PTMT-T cells depending on the cell number. (a) TNF-α. (b) IL-6. (c) IFN-γ. (d) Granulocyte-macrophage colony-stimulating factor (GM-CSF). Statistics, significance: The experiment was performed at least three times with replicates. Data are presented as mean ± SEM. *P*-values are calculated using an ANOVA. $^{*}p < 0.05$.

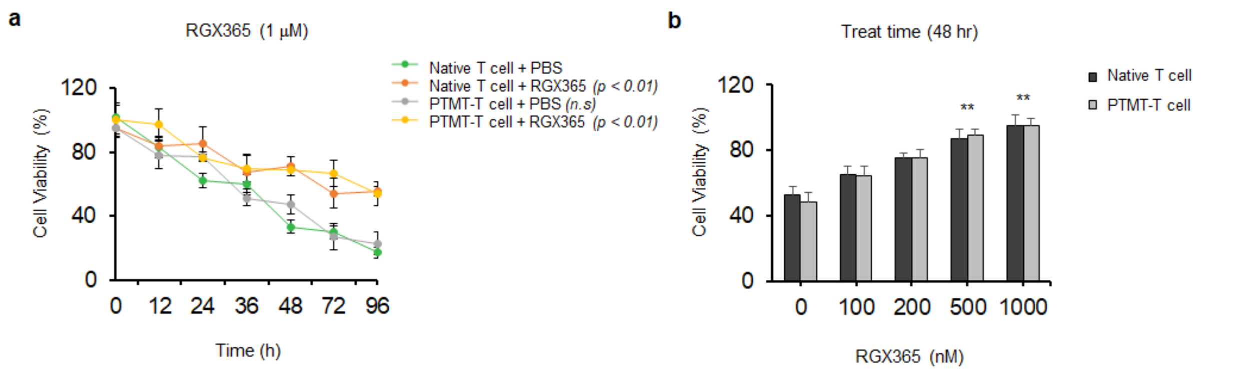

Figure S5. RGX365 as a cell preservation supplement. (a) Promoted viability of native and PTMT-T cells in the presence of RGX365. (b) Viability of native and PTMT-T cells depending on the concentration of RGX365. Statistics, significance: The experiment was performed at least three times with replicates. Data are presented as mean ± SEM. *P*-values are calculated using an ANOVA. $^{**}p < 0.01$.

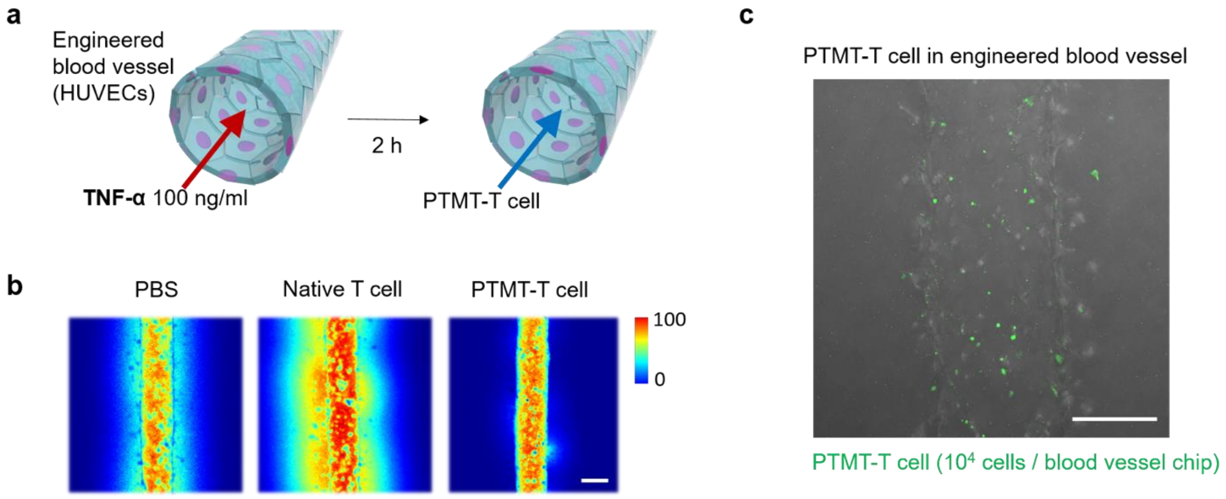

Figure S6. PTMT-T cell recover the vascular damage of engineered blood vessel. (a) Schematic illustration of experimental procedures using blood vessel-on-a-chip. TNF-α was utilized to disrupt the engineered blood vessel. (b) Recovery of damaged engineered blood vessels by PTMT-T cells. Scale bar, 200 µm. (c) Bright field image of engineered blood vessel merged with fluorescence images of PTMT-T cells. The PTMT-T cell was introduced into the perfusable blood vessel to regenerate TNF-α-mediated damage. Scale bar, 200 µm. The experiment was performed at least three times with replicates.

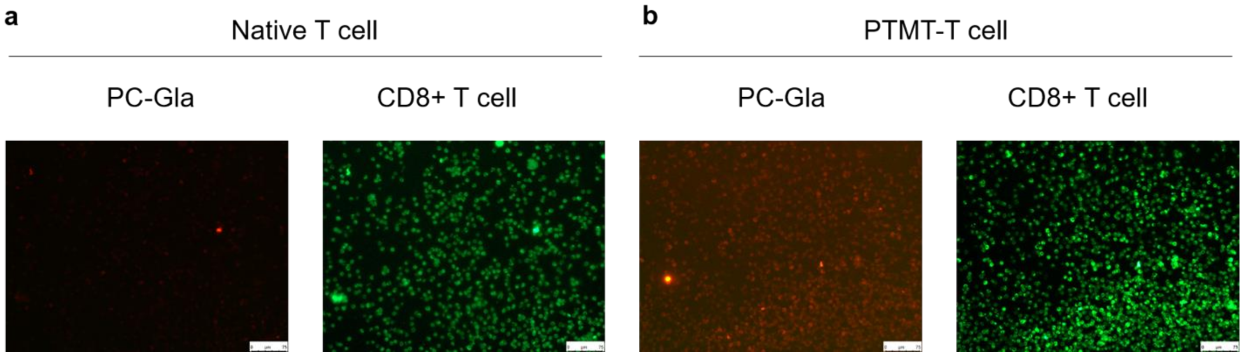

Figure S7. Expression of PC-Gla in the native and PTMT-T cells. (a) Native mouse CD8+ T cell. (b) Mouse CD8+ PTMT-T cell. Representative images from each group are shown (n = 5). Scale bar, 75 µm.

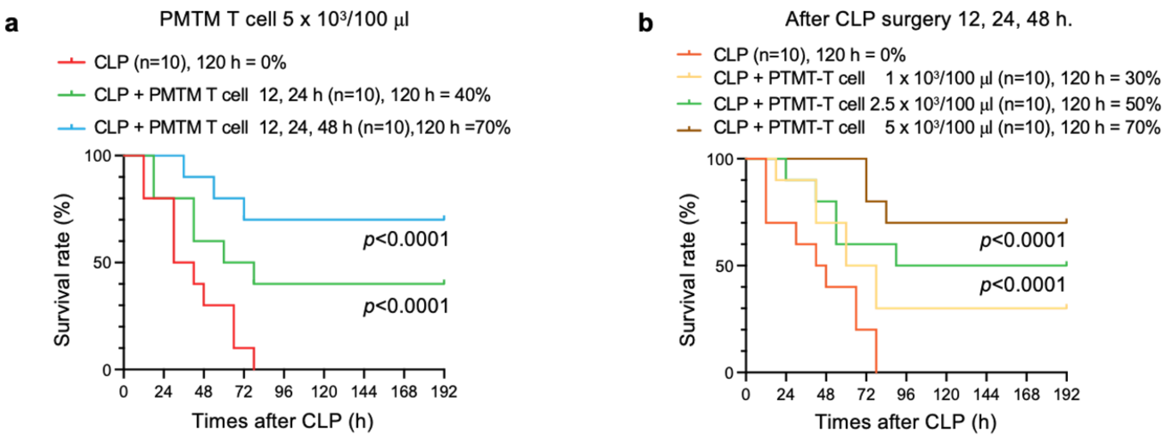

Figure S8. Survival rate of CLP-operated septic mice after the intravenous injection of PTMT-T cells (n = 10/ each group). (a) The effect of injection frequency in the survival rate of septic mice. (b) The effect of injected cell number in the survival rate of septic mice.

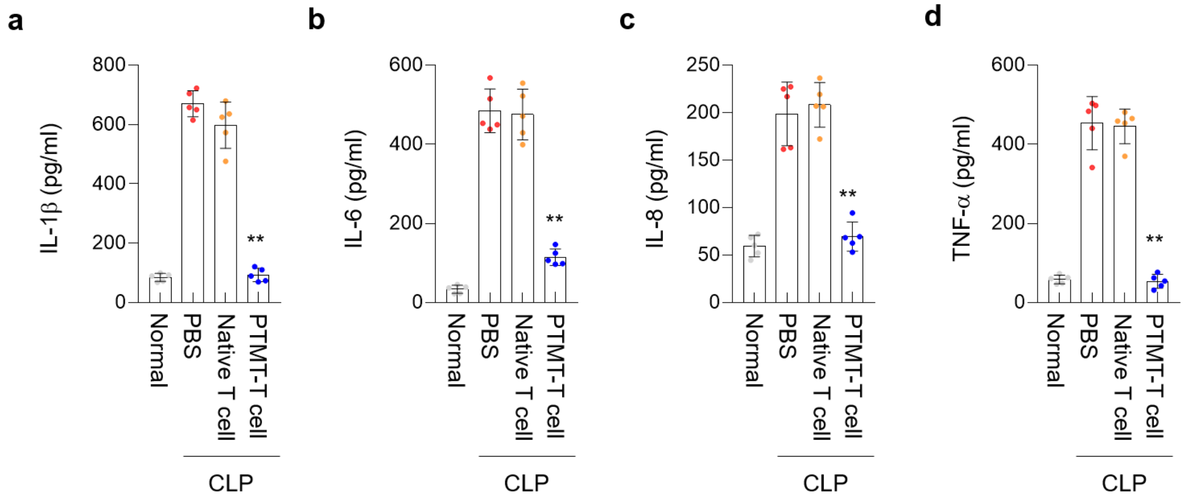

Figure S9. Serum cytokine levels in CLP-operated mice models after the treatment of PTMT-T cell. (a) IL-1β. (b) IL-6. (c) IL-8. (d) TNF-α. n = 5/each group. Statistics, significance: The experiment was performed at least three times with replicates. Data are presented as mean ± SEM. *P*-values are calculated using an ANOVA. $^{**}p < 0.01$.

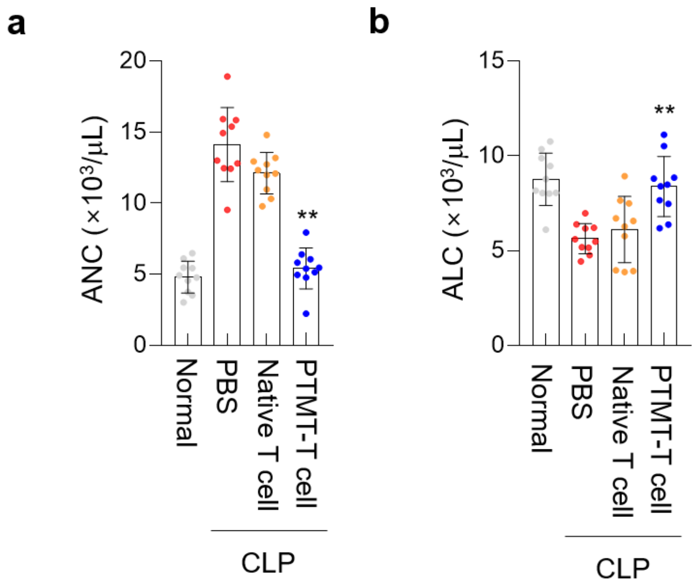

Figure S10. Changes of immune cell number in CLP-operated mice models after the intravenous treatment of PTMT-T cell. (a) Absolute neutrophil count (ANC). (b) Absolute leukocyte count (ALC). (**p<0.01). n = 5/each group. Statistics, significance: The experiment was performed at least three times with replicates. Data are presented as mean ± SEM. *P*-values are calculated using an ANOVA. $^{**}p < 0.01$.
